# Design of siRNA Therapeutics from the Molecular Scale

**DOI:** 10.3390/ph6040440

**Published:** 2013-03-25

**Authors:** Phillip Angart, Daniel Vocelle, Christina Chan, S. Patrick Walton

**Affiliations:** Department of Chemical Engineering and Materials Science, Michigan State University, 428 S. Shaw Lane, Room 2527, East Lansing, MI 48824, USA; E-Mails: angartph@msu.edu (P.A.); vocelled@msu.edu (D.V.); krischan@egr.msu.edu (C.C.)

**Keywords:** siRNA therapeutic, RNAi, liver cancer, siRNA design, delivery vehicle design

## Abstract

While protein-based therapeutics is well-established in the market, development of nucleic acid therapeutics has lagged. Short interfering RNAs (siRNAs) represent an exciting new direction for the pharmaceutical industry. These small, chemically synthesized RNAs can knock down the expression of target genes through the use of a native eukaryotic pathway called RNA interference (RNAi). Though siRNAs are routinely used in research studies of eukaryotic biological processes, transitioning the technology to the clinic has proven challenging. Early efforts to design an siRNA therapeutic have demonstrated the difficulties in generating a highly-active siRNA with good specificity and a delivery vehicle that can protect the siRNA as it is transported to a specific tissue. In this review article, we discuss design considerations for siRNA therapeutics, identifying criteria for choosing therapeutic targets, producing highly-active siRNA sequences, and designing an optimized delivery vehicle. Taken together, these design considerations provide logical guidelines for generating novel siRNA therapeutics.

## 1. Introduction

**Figure 1:**
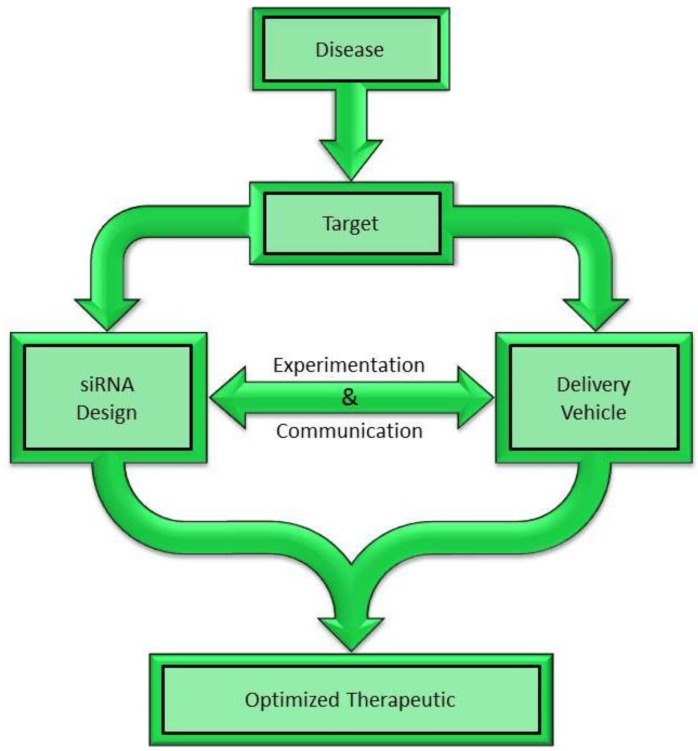
siRNA design overview. siRNA therapeutic design should, in the short term, begin by addressing diseases with unmet therapeutic need that are treatable by siRNA-based therapeutics. While initial siRNA and delivery vehicle design begins with some design heuristics, as discussed in the following sections, a completely optimized therapeutic will require concomitant, parallel development of each of the constituents followed by experimental validation of the optimized therapeutic.

The “big data” that is available and being generated on biological systems has greatly expanded the number of potential targets for treating disease. This is especially true for sequencing data yielding targets for proposed genetic therapies. As one such proposed therapy, siRNAs offer unique advantages that make them an ideal platform for therapeutic development. Perhaps the strongest argument in support of RNAi-based therapeutics is the ability to design the active agent, the siRNA, and its delivery vehicle simultaneously and mostly independent of each other for the knockdown of specific targets in specific tissues (Figure 1). Additionally, unlike protein-based biologics, little process development would be required for scale-up of siRNA-based therapeutics, as the synthesis and purification protocols for nucleic acids are well established. 

Effective silencing and the desired therapeutic effect depend upon the targeted delivery of an siRNA that is both specific and active against the intended target. Furthermore, the choice of an appropriate target largely determines the efficacy of an siRNA-based therapeutic. We therefore discuss the design of siRNA therapeutics in three broad categories: i.) selection of a disease and target mRNA, ii.) design of a highly-active siRNA, and iii.) design of the siRNA delivery vehicle. This review will discuss these aspects of siRNA therapeutic design and where relevant, use liver cancer to demonstrate key/fundamental design characteristics.

## 2. Choosing a Target for siRNA Mediated Silencing

Selecting a disease to target with any new therapeutic modality begins by choosing a disease for which current therapeutics do not work satisfactorily, thereby avoiding direct competition of the new therapeutic with existing, successful drugs. As the majority of drugs accumulate in the liver, diseases of the liver provide relatively easier targets for early-stage development of any therapeutic. With that in mind, we have chosen advanced liver cancer, specifically hepatocellular carcinoma (HCC), as an example for development of our hypothetical siRNA-based therapeutic. Liver cancer is the 6th most common cancer and the third leading cause of cancer related deaths, with ~750,000 new cases diagnosed annually worldwide [1]. Moreover, it has one of the highest rates of mortality, with only a 14% 5-year survival rate in the United States [2]. The high mortality rate highlights two needs, for earlier diagnosis of the disease and for improved treatments of advanced-stage liver cancer, the most common of which is hepatocellular carcinoma (HCC) [2]. The only approved treatment for advanced stage HCC is Sorafenib, a tyrosine kinase inhibitor having anti-angiogenic activity. Unfortunately, use of Sorafenib only increases median survival rate from 7.9 to 10.7 months over the placebo group [3]. While a number of clinical trials are currently underway using tyrosine kinase inhibitors, monoclonal antibodies, and one siRNA based treatment [4,5,6,7], continued development of therapeutics of all kinds for HCC is warranted.

For any disease, multiple proteins are potentially feasible drug targets. In order to select a particular protein target to knock down, it is useful to evaluate, if available, kinetic information about the transcriptional and translational regulation of the target under normal and diseased conditions. Typical siRNA-mediated knockdown only reduces protein levels to ~20% of baseline, and this effect is transient, lasting from a few hours to a couple of weeks before protein levels recover [8,9]. Thus, an siRNA therapeutic should only target mRNAs where this incomplete, transient knockdown is sufficient to achieve a therapeutic effect. To achieve a maximal therapeutic effect, it is also important to select a target where: (i) the protein half-life is shorter than that of the siRNA [8,10], (ii) the mRNA transcription rate is slower than the rate of siRNA-complex turnover [11], (iii) the concentration of mRNA is sufficiently low to permit silencing at minimal siRNA concentrations [12,13,14], and (iv) no feedback mechanism will upregulate transcription in response to the knockdown. Satisfying these conditions is possible through both, selecting appropriate target genes and engineering of siRNAs for maximal activity and stability. Engineering of the siRNA will be discussed in the next section.

In the development of an actual therapeutic, we would determine all of the necessary kinetic parameters experimentally. We would then select the target with the shortest protein half-life and confirm that its mRNA half-life is not extraordinarily short. Additional experiments that might be required would be to determine the basal levels of the mRNA targets (molecules/cell), as low expression level targets are more amenable to siRNA-mediated silencing. Using HCC as our example system, we examined five targets that have been previously identified and verified as HCC drug targets (Table 1). We were able to locate published values for protein and some mRNA half-lives [10,15,16,17,18,19,20]. VEGFR was found to have the shortest protein half-life, 70 min, and the known transcript half-lives were all between ~2–9 h [20], a small variability relative to the protein half-lives. Based on protein half-life alone, we concluded that VEGFR would be the most amenable HCC target for the development of an siRNA therapeutic. 

**Table 1:** Overview of some HCC targets.

Target	Gene Function	Current Clinical Drugs Targeting	Protein Half-life	References
VEGFR/VEGFR2	Angiogenesis	Sorafenib, Brivanib, Sunitinib, Cediranib, BIBF1120, Pazopanib, Regorafenib, E7080, TSU-68, Vandetanib, Ramucirumab, IMC-1121B	70 min	[5,15,21]
EGFR	Signal Transduction	Erlotinib, Cetuximab, Gefitinib, Lapatinib, Vandetanib	10 h	[5,16,21]
Bcr-ABL	Cell Proliferation	Destanib	40 h	[5,10,17,21]
MEK	Signal Transduction	AZD6244	6 h	[5,18,21]
PDGFR	Angiogenesis/Signal Transduction	Sorafenib, Brivanib, Linifanib, BIBF1120, Pazopanib, TSU-68	3 h	[5,19,21]

## 3. siRNA Design Considerations

siRNA mediated silencing possesses three characteristics that are desirable for a therapeutic modality: (i) it operates post-transcriptionally, (ii) the specificity of knockdown is high, and (iii) the same target mRNA can be inhibited by many different siRNA sequences. As siRNA sequences can have widely varying activities, significant effort has been spent identifying factors that enhance siRNA activity and reduce off-targeting and non-specific effects [22,23]. These features can be split coarsely into two categories, those related to siRNA activity and those related to siRNA specificity. siRNA activity is influenced by, among other factors, strand selection, the structure of the mRNA target region, base preferences, and overall siRNA G/C content. In comparison, siRNA specificity depends on strand selection, immunogenicity, and uniqueness of the target sequence. Designing an effective siRNA depends upon proper weighting of each of these factors; siRNA selection algorithms typically weight these factors based upon analyses of siRNA activity data [24]. As it stands, the rules for selecting active siRNAs continue to evolve, and it behooves the researcher to design siRNAs with features that are most strongly predictive of high activity. This section will discuss siRNA design considerations that are substantiated by biochemical evidence.

### 3.1. Details of the RNAi Mechanism

A canonical siRNA is a 21 nt duplex with 19 internal base pairs, 5’ phosphates, and dinucleotide 3’ overhangs [25,26,27,28,29,30]. When exogenous siRNAs enter the cytoplasm, they are first recognized by a protein complex called the RISC Loading complex (RLC), which is responsible for properly orienting the siRNA duplex as it hands off the guide strand to the active RNA Induced Silencing Complex (RISC) [30,31,32,33,34,35]. The RLC is a ribonucleoprotein complex minimally made up of Argonaute 2 (Ago2), Dicer, and TAR RNA Binding Protein (TRBP) [30,33,36,37,38]. The RLC orients the siRNA in the complex, and, in doing so, selects, sometimes incorrectly, one strand to serve as the guide strand that will target RISC to the mRNA [33,39,40,41,42]. The passenger strand (*i.e.*, the strand not selected by the RLC) is concomitantly cleaved and removed [33,43,44,45,46]. The active RISC then binds to its target mRNA by Watson-Crick base pairing. In a similar fashion to how the passenger strand is removed, RISC, specifically the Ago2 RNaseIII, cleaves the target at the center of the region complementary to the siRNA, resulting in degradation of the mRNA [28,42,47]. RISC is a multiple turnover enzyme that can then target other complementary mRNAs [42,46,47,48,49]. Additional details of the RNAi mechanism and siRNA-mediated silencing can be found in other sources [50,51].

### 3.2. Differential Terminal Hybridization Stability

For siRNAs to silence the intended target, they must be oriented by the RLC to ensure incorporation of the intended guide strand into the active RISC [30,33,37,38,40,46]. Incorporation of the unintended strand (*i.e*., the intended passenger strand) leads to the formation of a RISC that cannot cleave the intended target (reducing the activity of the siRNA therapeutic) and that can cause off-target effects (reducing the specificity of the siRNA) [39,40,52]. The difference in the activity of one strand relative to the other is termed *functional asymmetry* [40]. Functional asymmetry is a function of multiple factors, including RISC stability, RISC turnover rate, and, most directly, biased incorporation of one siRNA strand into RISC [33,48,53].

Biased strand incorporation occurs due to the recognition of differences in the termini of the siRNAs by the RLC proteins [33,37,38,39,40,54,55]. Based on early studies of functional asymmetry and biased strand loading [40], it was concluded that *differential terminal hybridization stability*, the difference in hybridization free energy between the two ends of the siRNA, was predictive of functional asymmetry due to biased strand loading [33,40]. These studies determined that the strand whose 5’ end is less stably hybridized (higher hybridization free energy) to the complementary strand is preferentially loaded into RISC [33,37,38]. Initially, differential hybridization stabilities were calculated using the four terminal base pairs at each end of the siRNA [40,56]. More recent work suggests that using only one nearest neighbor provides a more predictive calculation [54,57]. Nonetheless, when selecting candidate siRNAs, it is recommended to reject siRNAs with an unfavorable differential stability. The remaining candidates can then be pared down further using the sequence characteristics and other design criteria described below.

### 3.3. 5’ Nucleotide Preference

A number of positional base preferences, within the siRNA, are shown to be correlated to siRNA activity [22,58,59,60,61,62,63,64,65,66,67,68]. Most common among these are preferences affecting nucleotides at or near the 5’-termini of the siRNA strands [22,54,58,60,62,69]. To date, there is incomplete consensus among datasets as to what the most predictive base preferences are, nor is there strong biochemical evidence explaining the reason for specific base preferences. The exception is the two 5' terminal nucleotides [22,37,54,58,59,61,62,65,66], wherein Ago2 was identified as having a nucleotide specificity loop that shows significantly higher affinity interactions with U and A bases and lower affinity interactions with C and G bases [53]. By classifying the pool of siRNA strands according to their 5’-terminal nucleotides, the pool of candidates can be further limited to those sequences with the nucleotides that give the greatest likelihood of high activity [37,54]. 

### 3.4. mRNA Target Region

mRNA secondary structure precludes siRNA binding through steric hindrance of RISC binding and cleavage [70,71]. Conveniently, mRNA secondary structure can be easily, and relatively accurately, predicted *in silico*, using the nearest neighbor approach to predict the most thermodynamically stable structures [57,61,72,73,74,75]. If more certainty about the structure is desired/required, experimental structural analyses can be used to refine the predicted structures [71,76,77,78,79,80,81]. That said, the actual mRNA structure in a living cell is dynamic and any approach will provide only incomplete information that can guide choices of potential target regions. Using the available structural information, it is recommended, given a choice, to target regions of greater accessibility, especially at the 5’ and 3’ ends of the target region [75].

### 3.5. Immunogenicity

siRNAs can cause a number of immunogenic and cytotoxic responses, some of which arise due to their dsRNA structure and others that are sequence specific [82,83,84,85]. Immune receptors for siRNAs reside on the surface of the cell, within endocytotic vesicles, and within the cytosol [83]. While it is possible to prevent interaction of the siRNA with cell surface and endosomal receptors by protecting/inhibiting accessibility to the siRNA with a delivery vehicle (see section 4.1 below for additional details), it is still worthwhile to design siRNAs that avoid the use of immunostimulatory sequence motifs recognized by these receptors.

In general, cytosolic receptors for RNA are uniform in their expression across all cell types and recognize mainly structural features as opposed to sequence motifs [83]. Among these receptors are OAS1, PKR, and RIG-I [83,86,87,88,89,90,91]. The canonical siRNA structure does not activate PKR or RIG-1; these receptors are more sensitive to dsRNA longer than 30 bp or having 5’-triphosphates [87,89,92]. Unlike PKR and RIG-1, OAS1 is also activated by a sequence motif, NNWW(N_9_)WGN, in dsRNA as short as 19bp [86]. 

Cell surface and endosomal receptors for RNA, mainly the toll-like receptors (TLRs) [83,93], recognize specific sequence motifs, and their expression varies by cell type [83,94]. TLR3 (cell surface and endosomal) is a receptor for dsRNA [95,96]. TLRs 7 and 8 (endosomal) are receptors for ssRNA and are primarily responsible for recognition of specific RNA sequences, termed pathogen-associated molecular patterns (PAMPs) [94,96,97,98,99,100,101,102,103,104,105]. The recognition of siRNAs by TLR3 remains an area of investigation, as its activation was shown in clinical trials using naked siRNAs [103]. However, TLR3 *in vitro* was not activated by the canonical naked siRNA [94]. The recognition of siRNAs by TLRs 7 and 8 requires endocytosis of the siRNA followed by duplex melting within the acidic vesicle exposing the ssRNA bases to the TLR receptors, initiating an immune response [102,106]. When designing an siRNA, the immunostimulatory motifs to avoid include: GUCCUUCAA [107], UGUGU [98], UGU [98], UCA [108], GU-rich sequences (Heil 2004), AU-rich sequences [105], and U-rich sequences [102]. In addition to the immunostimulatory sequences, the UGGC motif has been shown to be cytotoxic via non-immune mechanisms [109].

### 3.6. Non-Specific Effects

Specificity of an siRNA begins with the selection of a sequence that is unique in the transcriptome of the target cells. However, sequences should also be chosen with consideration given to the possibility of off-target effects resulting from partial complementarity and miRNA-like targeting [84]. Repetitive sequences should also be avoided, in particular, the GGGG motif because it forms a G-quartet secondary structure [110]. 

miRNA-like targeting occurs when siRNAs have seed region complementarity with the 3’ UTR of an mRNA, resulting in translational repression of the untargeted transcript [111,112,113,114,115,116,117]. Moreover, mRNAs that are regulated by miRNAs are also more susceptible to miRNA-like off-targeting due to extended 3’ UTRs and the preexistence of miRNA target sites [118]. Avoiding miRNA-like targeting effects is complicated by an inability to accurately predict seed sequences [116,118,119]. miRNA-like targeting is influenced by the surrounding sequence of the target, the position of the target in the mRNA, and the repetitiveness of the target sequence [114,116,120,121]. As such the best way to account for miRNA-like targeting is to avoid seed sequences that have already been identified (http://www.mirbase.org) [122], so as not to unnecessarily limit the sequence space by avoiding false positives. A number of other tools exist that can predict off-targeting and may prove useful in determining the overall likelihood an siRNA will have strong off-target effects [123,124]. 

**Figure 2:**
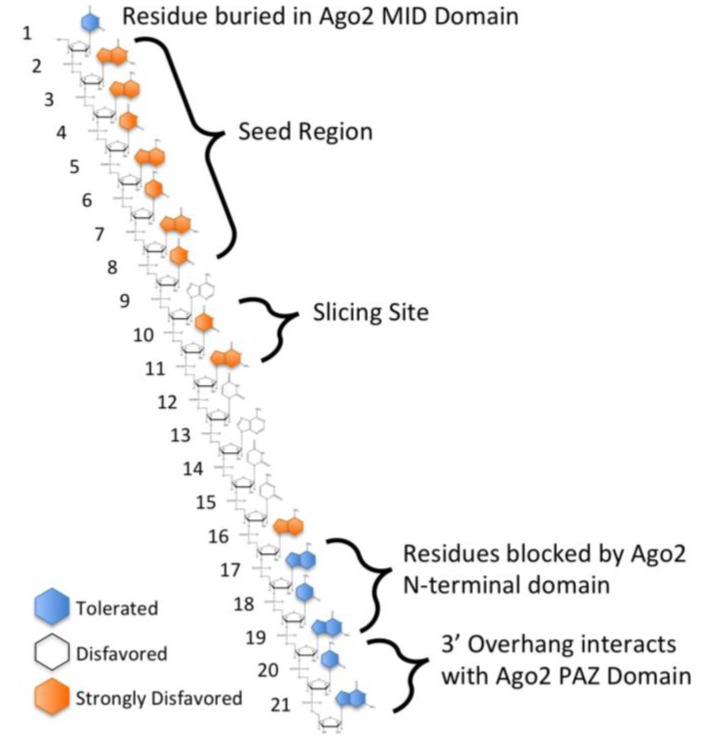
Tolerance for mismatches between an siRNA and its target. siRNA seed region (bases 2–8) [112,143] and splice site (bases 10–11) [127,132,144] are the least tolerant of mismatches because of their active role in silencing. The 1^st^ nucleotide [53,145], the nucleotides of the 3’overhang (bases 20–21) [146,147], and bases 17–19 [144,148] are the most tolerant of mismatches, because their ability to base pair is at least partially blocked by Ago2. Position and base specific mismatches are tolerated at positions 8–16 [141].

Methods to manipulate the siRNA to avoid off-target effects are not well established. However, there are a number of algorithms that do take off-targeting into account beyond a simple BLAST search [64,123,125]. Nonetheless, off-target effects are particularly challenging to avoid for a number of reasons. First, mismatches are tolerated within the duplex, however where mismatches are tolerated is only partially known (Figure 2) [126,127,128]. Second, predicting miRNA-like targeting is inaccurate and prediction of seed region interactions often leads to overestimation of off-targeting [118]. Lastly, siRNAs can cause translational repression, post-transcriptional silencing, or P-body based degradation and/or repression [24,129,130,131,132,133,134,135], making the cause of the off-target effects difficult to interpret. Still, as with immunogenicity, the only way to fully ensure that off-target effects do not occur is by post-hoc analysis using techniques such as RNA microarrays or parallel sequencing to quantify the levels of all untargeted transcripts [136,137,138] and antibody microarrays and mass spectrometry to check for off-target translational repression [139,140].

Genetic variation should also be considered, particularly single nucleotide polymorphisms (SNPs). Target regions with as little as one nucleotide difference can change target knockdown efficiency significantly [128,141]. Thus, in the short term, it makes sense to avoid targeting regions where SNPs occur to ensure the broad utility of any therapeutic. Going forward, the existence of inexpensive sequencing and synthesis technologies may allow the design of patient-specific siRNA therapeutics, accounting for each patient’s unique genotype.

From our perspective, the problem of avoiding non-specific effects reverts, largely, to the problem of designing an siRNA with the greatest specific activity. The most active siRNAs can be used at the lowest possible concentrations, decreasing off-target effects while still achieving a therapeutic effect [14]. Pooling siRNAs against a single target has been suggested as a means to improve specificity of silencing [142]. This effect is principally attributed to the reduced concentration of any given siRNA in the pool. However, we expect that design of the most active and specific RNA using guidelines such as those presented here will maximize the therapeutic window beyond what is possible by pooling. Thus, we would consider accounting for the factors described here as being of lesser importance relative to those that are predictive/indicative of the highest specific activity of a sequence.

### 3.7. Other siRNA Design Criteria

Other factors shown to influence siRNA activity include the G/C content (*i.e.*, the overall duplex stability) of the siRNA [22,39,149], the secondary structure of the guide strand [150,151], internal repeats [22], palindromic sequences [152], and positional base preferences along the siRNA [22,58,59,60,61,62,63,64,65,66,67,68,153]. More recently, additional structural criteria, even to the level of tertiary structure [154], have been identified as valuable in predicting siRNA activity. While each of these factors may be important in siRNA design, either their overall influence is thought to be minimal compared to the other selection criteria or there is a lack of consensus on how to implement the feature as a selection criterion. As such, selection of siRNAs using the mentioned criteria should be done as a second order set of rules to distinguish only the most active and specific siRNAs.

### 3.8. Non-Canonical siRNA Structural Designs

Using non-canonical siRNA structures in large part changes the point of entry into the RNAi pathway or changes the way in which the RNAi proteins interact with the non-canonical siRNA [155]. From the bottom up, ssRNAs are capable of being loaded by Ago2, albeit inefficiently, to form an active RISC *in vitro* [47,144,156,157] and *in vivo* when chemically modified [158,159]. By only providing one siRNA strand to enter RISC, proper strand selection is no longer a design characteristic. Other structures are designed to bias strand incorporation by altering the length of the passenger strand (asymmetric interfering RNAs (aiRNAs) and asymmetric short-duplex siRNAs (asiRNAs) [160,161,162]), the length of the 3’ overhang (fork-siRNAs (fsiRNAs) [163]), or by assembling a duplex with a segmented passenger strand (small internally segmented interfering RNAs (sisiRNAs) [164]). A more recently tested structure utilizes a “bulge” at the second position of the guide strand, creating a perturbation at the first base involved in the seed region [165]; the use of this modification to the canonical siRNA structure was shown to decrease off-targeting by miRNA-like activity. Other non-canonical structures exploit the ability for longer RNAs to enter RISC more efficiently; these structures are called Dicer substrate RNAs (DsiRNAs) [166,167,168,169]. 

### 3.9. Incorporation of Chemical Modifications

Chemical modifications in siRNAs can increase their stability (specifically with regards to nuclease degradation), minimize immunogenicity, and, to the extent possible, improve the activity of the siRNA [85,102,107,170,171,172,173,174,175]. Chemical modifications of the siRNA focus on changing the phosphodiester backbone, ribose sugar, nucleotide base, and 2'-OH ribose group. Effective use of chemical modifications requires the substitution of the new chemical moiety at a position within the siRNA where the additional group or structural alteration does not inhibit normal siRNA function. In general, the rationale behind chemical modifications is to incorporate small perturbations in the siRNA structure to prevent recognition and/or binding of the siRNA by nucleases and the immune receptors for RNA. The most common modifications include altering the 2'-OH group to a 2'-O-methyl or 2'-F to prevent recognition of the RNA by nucleases and TLR7 and TLR8 [85,173,176,177,178,179,180,181]. Unfortunately, no rules exist defining which chemical modifications are most useful and how they are best applied. 

The 3’ overhangs are also a common location for chemical modifications for two reasons: i) they may provide a site of attack for endoribonucleases and ii) chemical modifications, even bulky ones, are typically well tolerated at these positions [182]. Phosphorothioate and phosphorodithioate modifications to the backbone of the siRNA generate siRNAs with nuclease resistance, but the number and positions of modifications are important in retaining siRNA activity [176,183,184]. A more comprehensive understanding of the RNAi mechanism as well as the effects of various chemical modifications will aid in rational design of siRNAs with an even greater therapeutic index. A more extensive review discussing chemical modifications of siRNAs can be found elsewhere [185].

## 4. Delivery

**Figure 3:**
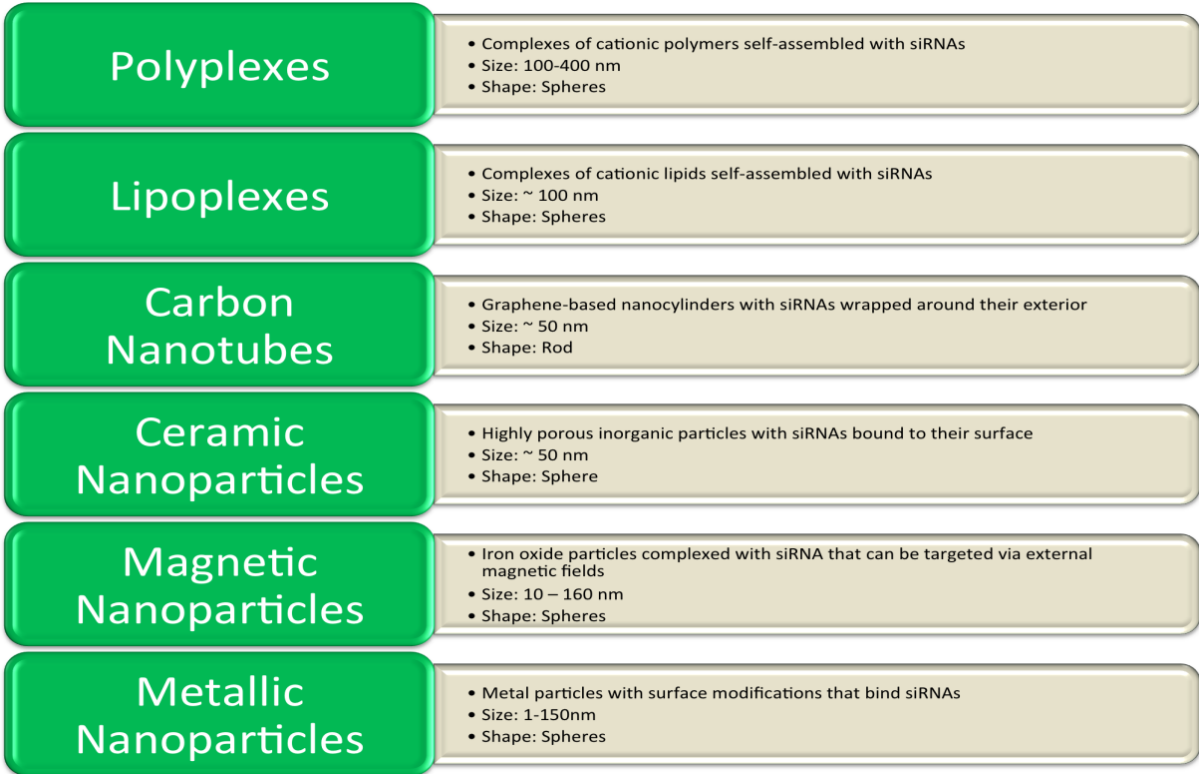
Types of non-viral delivery vehicles being utilized in developing siRNA therapeutics. For a more comprehensive review of each particle the following are suggested: Polyplexes [187,188,189], Lipoplexes [190,191,192], Carbon Nanotubes [184,193,194,195], Ceramic Nanoparticles [196,197,198], Magnetic Nanoparticles [199,200] and Metallic Nanoparticles [201,202,203]. Additional sources discussing the breadth of delivery vehicles that have been tested to date: [6,7,204,205,206].

siRNAs are hydrophilic, due to their anionic backbone, and do not readily diffuse across cellular membranes. Moreover, naked siRNAs are rapidly filtered from circulation, degraded, and can initiate immune/inflammatory responses (as stated above) [7,83]. Thus, delivery vehicles must be used to protect/conceal the siRNA while facilitating its transport to the cytoplasm of the targeted cells. By varying characteristics such as size, charge, shape, chemistry, and the means of directing the vehicle-siRNA complexes to the target cells, well-designed delivery vehicles can markedly increase the therapeutic efficacy of a given siRNA [186]. There are many different types of delivery vehicles that have been developed for siRNA-based therapeutics, with lipoplexes and polyplexes currently being the most commonly applied, each possessing different physical and chemical characteristics, as well as offering distinct advantages and disadvantages (Figure 3). The following sections describe a design approach in which the desired properties of the delivery vehicle are selected based on the unique characteristics of the targeted disease. As an illustrative example, these guidelines are applied to the development of a hypothetical HCC therapeutic. The following sections describe these various vehicle types and outline approaches for the design of an siRNA delivery vehicle. 

### 4.1. Accessing the Cell Cytoplasm

The purpose of the delivery vehicle is to protect the siRNA and deliver it to the cytoplasm of the target cells where it can be recognized by the proteins of the RNAi pathway. To reach the cytoplasm, vehicle-siRNA complexes can, as is the case with many lipoplexes, cross the membrane directly [207] or be internalized by endocytosis followed by escape from the vesicle, often by vesicle rupture [208,209,210]. Vehicle-siRNA complexes are formed in two ways: self-assembly by electrostatics (typical) and covalent attachment (rare). For self-assembled complexes, siRNAs attach on the surface or are fully encapsulated depending on the structure and flexibility of the vehicles. The fully formed complex reduces siRNA degradation by serum nucleases, recognition by toll-like receptors (TLRs), and other molecules of the innate immune system [83]. siRNAs that are not fully encapsulated are more likely to be recognized by serum nucleases and TLRs. While the affinity of the vehicle for the siRNA is critical for complex formation, the complex must dissociate upon cell entry to allow the siRNA to be bound by the pathway proteins [211].

The various endocytotic mechanisms take up species of particular sizes as follows: lipid rafts (40–50 nm), caveolae-mediated endocytosis (50–60 nm), clathrin-mediated endocytosis (<200 nm), macropinocytosis (500–10,000 nm, and phagocytosis (<10,000 nm) [212,213]. Current data suggests that delivery leading to siRNA silencing can occur through multiple uptake mechanisms depending on the vehicle type [186,214,215]. Additionally, all endocytotic pathways efficiently incorporate complexes with a net positive charge [216].

When taking these factors into account, the endocytotic pathways used most frequently by the diseased cells will dictate both the characteristics of the chosen delivery vehicle. For our hypothetical case of liver cancer, HCCs primarily use pinocytosis for the uptake of extracellular components. As such, our vehicle-siRNA complexes should have a maximum diameter of 10,000 nm and a net positive charge [217]. These design requirements do not eliminate any candidate vehicle types (Figure 3) from potentially being used in a therapeutic for the treatment of HCCs.

### 4.2. Routes of Systemic Delivery

Each tissue is connected to multiple transport systems (e.g., circulatory, lymphatic, *etc.*). Delivery to the intended target can be enhanced by pairing the characteristics of a therapeutic complex to the characteristics of the transport system that will deliver the complex to the target cells. [218,219]. The goal then becomes to maximize the residence time of the therapeutic complex in a given transport system before it is degraded or sequestered. For diseases in tissues that are difficult to access by transport systems, e.g., prostate, eye, neck, and brain, localized delivery is often more appropriate [205].

The circulatory system is the most common route for therapeutic delivery. In the circulatory system, particles are actively filtered based on size. Particles less than 10 nm in size are filtered by the kidneys within hours of injection, and particles larger than 200 nm are retained within the spleen [220,221]. Thus, to improve the bioavailability of the complexes, it is optimal to design complexes to between 50–200 nm [222].

Bioavailability can be further improved by avoiding recognition and clearance of the complexes by phagocytes. While particle size can be manipulated to avoid phagocytosis, the size range and how much size changes affect clearance differ among particle types [223]. Shape has been shown to have a more consistent effect, with shapes containing large tangential planes (e.g., rods) being phagocytosed more efficiently than particles of uniform diameter (e.g., spheres) [224,225,226]. Complexes of high zeta potential (>25 mV) are also phagocytosed more efficiently than complexes with potential below 15 mV [227]. PEGylation and the use of hydrophilic polymer coatings have also been shown to inhibit phagocytosis [228,229,230]. 

Based on the intent to reach HCCs in the liver as well as metastatic cells, the cardiovascular system provides the best system for delivery. Bioavailability within the system is thus optimized using spherically shaped complexes, 50–200 nm in diameter, with a 0 to 15 mV zeta potential, with PEGylation as needed to achieve the desired circulating half-life. These complex characteristics would reduce clearance from circulation, increasing their access to HCCs.

### 4.3. Delivery Specifically to Target Cells

Having designed the complex for maximal bioavailability, consideration must now be given to trafficking out of the transport system to the target cells. For instance, tumors, including HCC, are susceptible to enhanced permeability and retention (EPR) which results in an increased uptake and sequestration of particles 100–500 nm in size from the circulation ("passive" targeting) [231,232,233]. Designing the size of complexes to be 100–200 nm will result in enhanced accumulation in the vicinity of an HCC tumor, without compromising other design characteristics.

Delivery vehicles can also be modified with surface ligands (e.g., peptides, antibodies, or aptamers) that guide and aid uptake by the target cells ("active targeting") [234]. While easily applied for complexes with encapsulated siRNA, complexes with surface attached siRNA will have reduced surface area for siRNA attachment with the addition of each ligand. Active targeting has proven effective in HCC models [235,236,237], but the strategies for functionalizing delivery vehicles with ligands are not well defined. In situations where targeting cannot be improved through molecular approaches, magnetic nanoparticles can be directed to a specific target region via external magnetic fields, though this can be logistically difficult [200].

### 4.4. Methods of Administration

Multiple approaches can be applied for local or systemic administration to the body. Current therapeutics favor localized delivery (intranasal, intraocular, intratumoral, *etc.*) for their specificity in reaching a target tissue [238], though this is difficult for relatively remote tissues where the disease may be systemic, as for metastatic HCC. In such cases, the circulatory system can be used to access cells at nearly any location in the body, with the possible exception of the brain due to the blood-brain barrier. For access to the circulatory system, transdermal and gastrointestinal absorption can be used, but intravenous (IV) injection is optimal for the immediate delivery of known concentrations of a therapeutic [239]. For IV therapeutics, complexes should be designed to be stable for long-term storage in a saline or intravascular compatible solution. While systemic delivery always has the potential to allow non-specific effects outside the target tissue, these effects can be mitigated through the inclusion of cell-specific targeting moieties that limit uptake by non-targeted cells while not preventing access to targeted cells at remote locations.

### 4.5. Selection of a Specific Delivery Vehicle for Targeting HCCs

**Figure 4:**
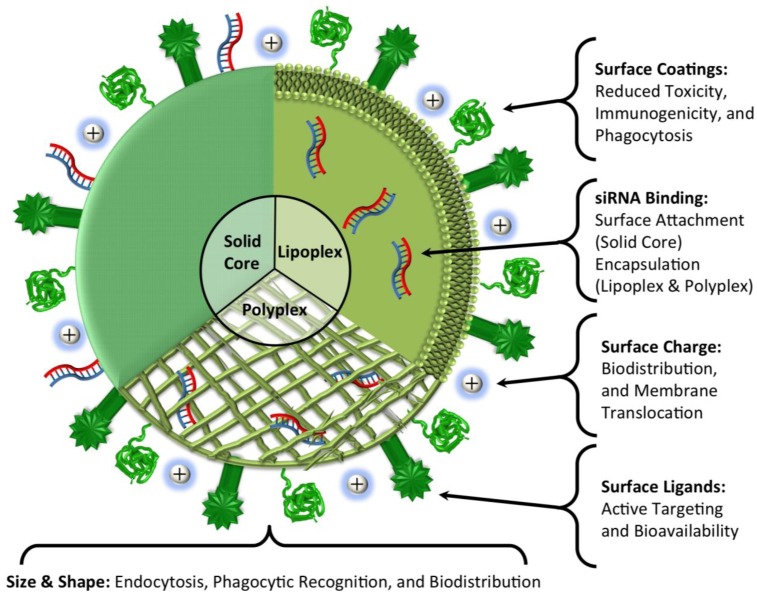
Design characteristics of vehicle-siRNA complexes. A variety of characteristics can be manipulated to improve the bioavailability and biodistribution of vehicle-siRNA complexes while reducing their cytotoxicity and immunogenicity.

Each of the variety of delivery vehicle types has unique characteristics that are advantageous for particular applications. However, some vehicle types may not perform as well as others in our case of targeting HCCs. Carbon nanotubes are readily phagocytosed [195]. Ceramic, magnetic, and metallic delivery vehicles have issues with long term accumulation and cytotoxicity [198,201]. Thus, the delivery vehicles most likely to be successful for this application are lipoplexes or polyplexes (Figure 4) [207,235,239,240].

Further comparison of these two platforms can be based on available experimental data, in particular published results from clinical trials. One type of lipoplex, stable nucleic acid lipid particles (SNALPs), is currently being used in 6 of 10 clinical trials for systemic delivery of siRNAs and has achieved therapeutic efficacy at low doses *in vivo* (ED_50_ = 0.3 mg/kg) [6,7,190,191,241]. Cytotoxicity and pulmonary inflammation related to SNALPs raise concerns for their therapeutic application and has been the result of clinical trials being terminated [242,243]. Polyplexes are currently being applied in one clinical trial for the systemic delivery of siRNA with therapeutic effects at ED_50_ = 2 mg/kg *in vivo* and TD_50_ = 27 mg/kg *in vivo* [244,245,246]. Recent studies have shown that biodegradable bonds within the polymer have further reduced toxicity and improved polyplex residence time due to decreased recognition by phagocytes [187,247,248,249], even though, no toxicity was observed in clinical trials [244]. The choice between a lipoplex or polyplex platform reduces to the preference of increased delivery efficiency (lipoplexes) or reduced toxicity (polyplexes).

## 5. Conclusions

Current data permits mechanism-driven design of both siRNAs and their delivery vehicles, at least to some extent. Perhaps one of most exciting aspects of siRNA design is the ability to target so many different disease states once an effective delivery vehicle exists for a given tissue type, as exemplified by the SNALP and polyplex platforms currently in clinical trials. In the future, the "plug and play" nature of these combinations of siRNA and vehicle could potentially support the development of personalized therapeutics on a patient by patient basis. Still more comprehensive methods for siRNA sequence selection and modification will be driven by a more complete understanding of the RNAi mechanism. Continuing advances in vehicle chemistry and careful analysis of vehicle-siRNA complex structures will improve the specificity and efficiency of siRNA therapeutics.
